# Comment on Sobczyk, M.K.; Gaunt, T.R. The Effect of Circulating Zinc, Selenium, Copper and Vitamin K_1_ on COVID-19 Outcomes: A Mendelian Randomization Study. *Nutrients* 2022, *14*, 233

**DOI:** 10.3390/nu14051112

**Published:** 2022-03-07

**Authors:** Rob Janssen, Cees Vermeer, Jona Walk, Allan Linneberg

**Affiliations:** 1 Department of Pulmonary Medicine, Canisius-Wilhelmina Hospital, 6532 SZ Nijmegen, The Netherlands; 2 Cardiovascular Research Institute CARIM, Maastricht University, 6229 ER Maastricht, The Netherlands; cees.vermeer@outlook.com; 3 Department of Internal Medicine, Canisius-Wilhelmina Hospital, 6532 SZ Nijmegen, The Netherlands; Jona.Walk@cwz.nl; 4 Center for Clinical Research and Prevention, Bispebjerg and Frederiksberg Hospital, DK-2000 Copenhagen, Denmark; allan.linneberg@regionh.dk; 5 Department of Clinical Medicine, Faculty of Health and Medical Sciences, University of Copenhagen, DK-2200 Copenhagen, Denmark

Sobczyk and Gaunt genetically predicted circulating zinc, selenium, copper, and vitamin K_1_ levels—instead of directly measuring nutrients in blood—and hypothesized that these levels would associate with SARS-CoV-2 infection and COVID-19 severity [1]. We have concerns about their conclusions regarding vitamin K in COVID-19. Major study limitations were that the genetic instruments had not demonstrated reliable association with the measured exposure (plasma vitamin K_1_) and that the authors used the same genome-wide association study for instrument discovery and effect estimation. Moreover, even direct quantification of blood vitamin K_1_ concentrations is not a valid method for quantifying vitamin K_1_ status, since this assessment only reflects a snapshot of recent vitamin K_1_ intake, is sensitive to triglyceride concentrations, and gives little information about the vitamin K_1_ utilization in tissue.

There are also differences between vitamins K_1_ and K_2_ in half-life time, tissue distribution, and bioavailability [2]. Vitamin K_2_ has a much longer half-life and may, therefore, be important particularly during acute illness, where vitamin K reserves are being used and become less available in peripheral tissues. Consumption of vitamin K_2_ is usually too low to accurately quantify their plasma concentration. Due to these factors, most experts in the field advocate measuring levels of inactive circulating vitamin-K-dependent proteins to assess the combined deficiency of vitamins K_1_ and K_2_. In our studies, we used *PIVKA*-*II* and *dp*-*ucMGP* as measures of hepatic and extrahepatic vitamin K status, respectively [3,4,5]. Particularly extrahepatic vitamin K status is severely compromised in COVID-19, and high dp-ucMGP levels are associated with increased mortality [4,5]. 

Another debatable assumption made by Sobczyk and Gaunt is that the baseline vitamin K status—at the moment of SARS-CoV-2 contraction—is a predictive factor for the disease course of subsequently developing COVID-19 [1]. An alternative explanation for the poor vitamin K status in COVID-19 patients is high vitamin K expenditure during the disease. Interestingly, observations in individuals using vitamin K antagonists as anticoagulant drugs support our theory that it is mainly increased vitamin K utilization during the infection, rather than poor baseline vitamin K status, that is responsible for the extrahepatic vitamin K deficiency we found in our studies [6,7]. 

Given that Sobczyk and Gaunt may not have accurately predicted overall extrahepatic vitamin K status, and that they estimated pre-COVID rather than vitamin K levels during the infection, we are of the opinion that their genetic data analysis is interesting but cannot be used to decide whether vitamin K supplementation has a role in COVID-19.
